# Detection of *Pratylenchus zeae* and *P. brachyurus* parasitizing plants from the caatinga biome, Ceará, Brazil

**DOI:** 10.21307/jofnem-2021-019

**Published:** 2021-02-25

**Authors:** Francisco Jorge Carlos Souza Junior, Mayara Castro Assunção

**Affiliations:** Departamento de Agronomia, Universidade Federal Rural de Pernambuco, Dois Irmãos, 52.171-900, Recife, PE, Brazil

**Keywords:** *Pratylenchus zeae*, *Pratylenchus brachyurus*, Molecular biology, Morphology, Root lesion nematode, Caatinga plants

## Abstract

Roots of plants characteristic of the Brazilian caatinga showing necrosis symptoms were observed in Iguatu, CE, Brazil. To identify the species, morphological characterization was performed, through the morphometry of females, and molecular analysis of the ITS and 28S rDNA regions. The nematodes *Pratylenchus zeae* and *P. brachyurus* were identified as causal agents, confirming pathogenicity by Koch postulates. This is the first report of *P. zeae* and *P. brachyurus* in caaatinga plants in the state of Ceará, Brazil.

The caatinga is an exclusively Brazilian biome, occupying about 10% of the national territory, which covers various plant types in the Northeast region and extends to several states in the country. In Ceará, this biome occurs in 100% of the state, being characterized by a semi-arid climate with extreme conditions: high solar radiation, low relative humidity, little precipitation, and low cloudiness (Araújo et al., 2007; Instituto Brasileiro de Geografia e Estatística (IBGE), 2020; Prado, 2003).

The caatinga biodiversity comprises over 900 species of plants, including *Cereus jamacaru* D. C., *Myracrodruon urundeuva* Fr. All., *Caesalpinia pyramidalis* Tul. and *Pilosocereus gounellei* (A. Weber ex K. Schum.) Bly. ex Rowl.) are well known and common to be found, exercising importance in maintaining the native vegetation of this biome (Ministério do Meio Ambiente (MMA), 2012). However, the caatinga is significantly altered, approximately 36% has been modified by man, being replaced by pastures and agriculture that bring with it the introduction of phytopathogenic organisms, such as nematodes, which cause imbalance in the original microbiota (Instituto Brasileiro de Geografia e Estatística (IBGE), 2020).

Nematodes are organisms that live in the soil and can be found parasitizing the root system, causing direct damage to plants. One of the main genres is *Pratylenchus* Filipjev (1936) is known as a nematode of root lesions, being disseminated in several geographical locations, occupying the third position in the world ranking of nematodes harmful to agriculture, it presents a wide range of hosts in economically important cultures (Jones et al., 2013). In Brazil, it has a wide distribution, present in all regions, also parasiting native plants, which can act as alternative hosts and source of inoculum (Gonzaga et al., 2016).

In August 2019, samples of roots and rhizospheric soil were collected in caatinga areas of the plants of *Cereus jamacaru*, *Myracrodruon urundeuva*, *Caesalpinia pyramidalis*, and *Pilosocereus gounellei* in the municipality of Iguatu, CE (6° 25′18.7″S 39° 19′57.8″W). The samples were processed to extract the nematodes from the roots (Coolen and D´Herde, 1972) and soil (Jenkins, 1964).

For morphological characterization, the specimens were killed in a water bath (55°C) and fixed in formalin-acetic acid (Goodey, 1957). The morphometric characteristics and De Man (1880) (V%, *a, b, b′, c*, and *c′*) were obtained from adult females (Castillo and Vovlas, 2007).

The molecular identification of specimens from the population of *Pratylenchus* was carried out by amplifying and sequencing the regions ITS primers with VRAIN2F (5´-CTTTGTACACACCGCCCGTCGCT-3´) and VRAIN2R (5´-TTTCACTCGCCGTTACTAAGGGAATC-3´) (Vrain et al., 1992) and D2-D3 of 28S rDNA segment with the primers D2A (5´-ACAAGTACCGTGAGGGAAAGTTG-3´) and D3B (5´-TCGGAAGGAACCAGCTACTA-3´) (De Ley et al., 1999).

The consensus sequences were formed from the forward and reverse sequences, using the Staden package (Staden et al., 1998). All consensus sequences obtained were used to compare with the NCBI nucleotide database, based on the research using the blastn algorithm. Several sequence alignments for each individual gene were generated with the online version of MAFFT version 7 with the iterative refinement method L-INS-i (Katoh, 2013; Katoh and Toh 2008). Phylogenetic analysis used the maximum likelihood (ML) methods for individual genes, performed via RAxML-HCP2 v.8.2.8 (Stamatakis, 2014) implemented in CIPRES Portal v.2.0 (https://www.phylo.org/portal2/home.action) with 1,000 repetitions in the GTR-GAMMA model.

According to the morphological and molecular characterization, two species of *Pratylenchus* were identified: *P. zeae* and *P. brachyurus*, with a predominance of *P. zeae*, corresponding to 80% of all specimens, and *P. brachyurus* with only 20% of the total isolates. *P. zeae* was identified in all hosts, *P. brachyurus* was only identified in *M. urundeuva*.

The females (*n* = 30) of *P. zeae* showed the stylet measured 17.2 ± 0.25 (17.10-17.98) μm; vulva position was 71.60 ± 1.31 (71.95-72.55)% of body length; *c* = 17.78 ± 0.44 (17.57-18.59) μm and *c′* = 2.33 ± 0.18 (2.09-2.42) μm. Total body length was 630.8 ± 30.36 (601.21-690.60) μm, with *a* = 23.98 ± 1.76 (21.30-25.34) μm, *b* = 8.1 ± 1.91 (7.17-9.00) μm, and *b′* = 3.42±0.11 (3.16-3.61) μm. *P. brachyurus* females (*n* = 30) showed the stylet measured 19.61 ± 0.71 (18.21-20.81) μm; vulva position was 84.48 ± 1.74 (83.58-86.30)% of body length; *c* = 19.26 ± 1.05 (18.87-20.51) μm and *c′* = 2.10 ± 0.33 (1.84-2.52) μm. Total body length was 535.33 ± 21.58 (508.63-562.70) μm, with *a* = 24.47 ±1.37 (23.36-27.91) μm, *b* = 7.73 ± 1.01 (6.14-9.50) μm, and *b′* = 4.22 ± 0.53 (3.67-5.15) μm.

The sequences of the studied rDNA regions were submitted to GenBank (ITS: MT994745, MW350685-MW350688 and D2-D3 28S: MT994748, MW349659-MW349662). The populations CFN001, CFN003, CFN004, and CFN005 used for molecular analysis showed a high degree of sequence identity (99%) with *P. zeae* from Brazil and China for the ITS and D2-D3 28S region. The CFN002 population showed coverage of 98% homology and 99% in consultation with *P. brachyurus* sequences from Brazil and Kenya for the ITS and D2-D3 28S region (Figs. 1, 2).

**Figure 1:**
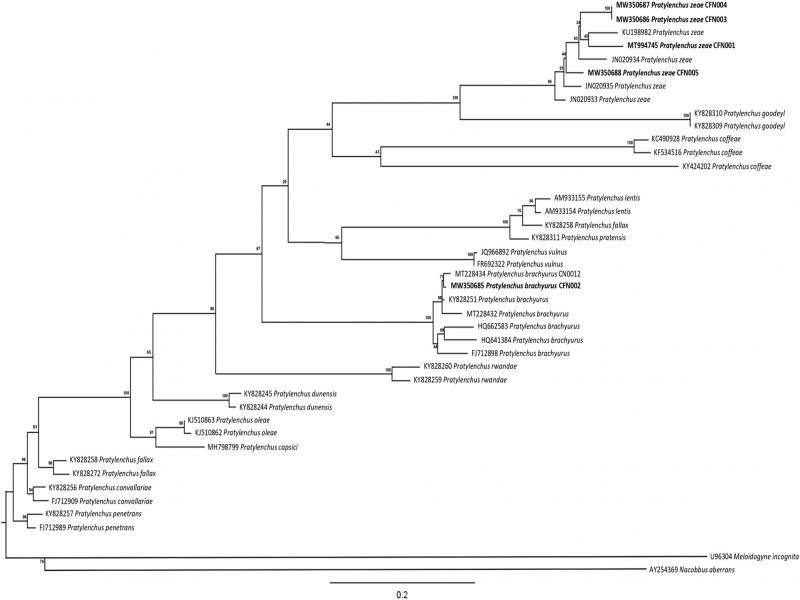
Tree of the phylogenetic relationships of *Pratylenchus zeae* and *P. brachyurus* and populations with other *Pratylenchus* spp. as inferred from the maximum likelihood analysis of ITS. *Nacobbus aberrans* and *Meloidogyne incognita* were used as outgroups. The scale bar indicates the expected number of substitutions per site.

**Figure 2:**
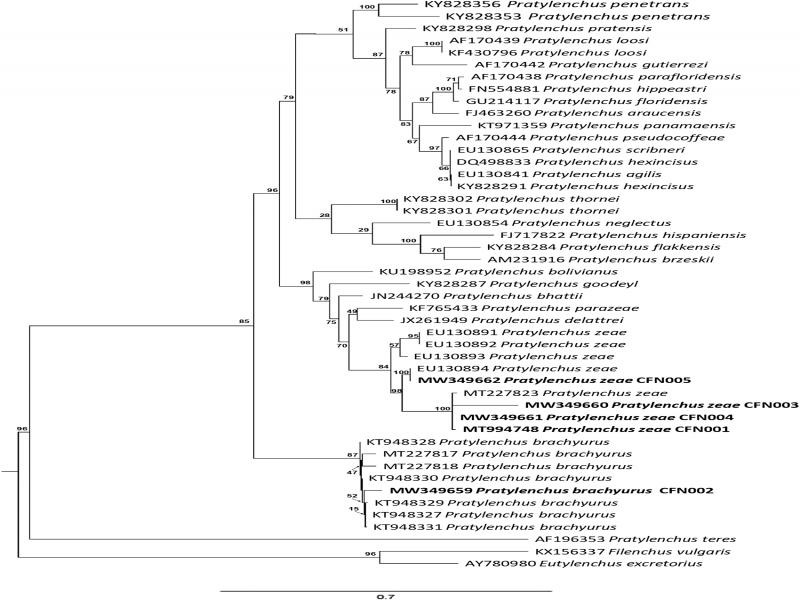
Tree of the phylogenetic relationships of *Pratylenchus zeae* and *P. brachyurus* and populations with other *Pratylenchus* spp. as inferred from the maximum likelihood analysis of 28S rDNA. *Eutylenchus excretorius* and *Psilenchus vulgaris* were used as outgroups. The scale bar indicates the expected number of substitutions per site.

The Koch postulates were performed separately for *P. zeae* and *P. brachyurus*. The populations of *P. zeae* and *P. brachyurus*, obtained in the caatinga field of the analyzed plant species, were kept in okra ‘Santa Cruz’ in pots under greenhouse conditions and, subsequently, the infected roots of the okra were processed for the extraction of eggs and second stage juveniles (J2) for inoculation.

Plants of *Cereus jamacaru*, *Myracrodruon urundeuva*, *Caesalpinia pyramidalis*, and *Pilosocereus gounellei* were kept in pots containing soil previously sterilized in a greenhouse (average temperature of 25.5 ± 1°C) and were inoculated with 5,000 eggs and J2 of *P. zeae* and *P. brachyurus* per pot. Inoculation was carried out by placing the inoculum suspension in holes approximately 4 cm deep in the rhizosphere of each plant. The experimental design used was completely randomized, with five times per plant species plus five control plants, which were not inoculated. Inoculated plants exhibited root necrosis symptoms similar to those observed in the field, while uninoculated plants showed no symptoms.

This is the first report of *P. zeae* and *P. brachyurus* parasitizing roots of *Cereus jamacaru*, *Caesalpinia pyramidalis*, and *Pilosocereus gounellei* in Brazil. However, in 2013, *Pratylenchus* spp. associated with *Myracrodruon urundeuva* in the state of Minas Gerais (Favoreto et al., 2013), but this research makes the first mention of this two nematode (*P. zeae* and *P. brachyurus*) for this plant in the Northeast, the main niche of the caatinga biome.

In this way, this research brings a new data about the parasitism of the genus *Pratylenchus* in plants in the caatinga biome, updating fundamental elements for the elaboration of the host-pathogen relationship and its interference in the maintenance of native plants in this biome in the state of Ceará.
